# Global Trends in Research of Brain‐Computer Interfaces in Neuroscience From 2014 to 2023: A Bibliometric Analysis

**DOI:** 10.1002/cns.70851

**Published:** 2026-03-27

**Authors:** Yangfan Yu, Wentong Liu, Sirui Ju, Lishan He, Nanting Chen, Alexandr N. Chernov, Jianfeng Zhang, Jinning Mao, Guodong Liu

**Affiliations:** ^1^ Chongqing Medical University Chongqing China; ^2^ Institute of Experimental Medicine Saint Petersburg Russia; ^3^ Yunyang County People's Hospital Chongqing China; ^4^ The Second Affiliated Hospital of Chongqing Medical University Chongqing China

**Keywords:** bibliometrics, brain‐computer interfaces, CiteSpace, neuroscience, VOSviewer

## Abstract

**Aim:**

Brain‐computer interfaces (BCIs) represent a promising technology for addressing neurological disorders, with growing research interest globally. This study aimed to map global research trends in BCI neuroscience from 2014 to 2023 via bibliometric analysis, identifying key contributors and hot topics to inform future research.

**Methods:**

A total of 2386 publications related to BCIs in neuroscience were retrieved from the Web of Science Core Collection. Bibliometric analyses, including co‐authorship networks, keyword co‐occurrence, and burst detection, were performed using VOSviewer, R, and CiteSpace. The study analyzed publications by country, institution, journal, author, and keyword to map the landscape of global research activity.

**Results:**

China emerged as the country with the highest number of publications, and the International Journal of Neural Engineering was the most productive journal. Co‐authorship analysis revealed collaborative networks across global institutions, while keyword co‐occurrence and burst detection identified electroencephalography (EEG), rehabilitation, and motor cortex as the most prominent research hotspots in recent years.

**Conclusion:**

This analysis provides a reference for researchers and data support for future studies, clarifying the global landscape and priorities in BCI neuroscience research.

## Graphical abstract

This bibliometric study summarizes the global progress of brain‐computer interfaces (BCIs) research based on 2386 publications from 2014 to 2023. Our analysis identifies China as a leading contributor, with electroencephalography (EEG), rehabilitation, and motor cortex emerging as key research hotspots, providing important guidance for future BCI development.

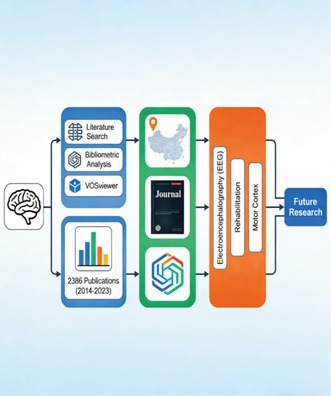

## 1 Introduction

Brain‐computer interfaces (BCIs) are disruptive technologies integrating multiple frontier disciplines, including neuroscience, biomedical engineering, computer science, and artificial intelligence [1, 2]. Essentially, BCI systems acquire electrophysiological or metabolic signals from brain activity via specialized sensors, apply advanced signal processing and pattern recognition algorithms for real‐time decoding, and translate user intent into executable commands [3]. This transformative capability provides groundbreaking solutions for patients with severe functional impairments [4]. Current neurorehabilitation applications encompass brain‐controlled wheelchairs [5], neuroprosthetics [6], and communication devices [7]. These BCI implementations not only enhance patients' quality of life but also significantly alleviate the burden and costs for family members and caregivers.

The primary signal acquisition modalities for BCIs encompass non‐invasive (EEG/fNIRS), semi‐invasive (ECoG), and invasive approaches (Microelectrode Arrays), each enabling distinct application scenarios. Invasive BCIs can record signals with high spatiotemporal resolution, demonstrating superior performance in decoding complex motor tasks [8]. In 2025, Stanford researchers developed a 4‐degree‐of‐freedom (4‐DOF) finger decoding system using microelectrode arrays implanted in the left precentral gyrus, achieving dexterous control comparable to natural hand motions—a substantial advancement beyond the single‐dimensional control constraints of conventional BCIs [9].

The rapid expansion of BCI research, coupled with its diverse technological approaches and inherent interdisciplinarity, necessitates a systematic bibliometric analysis of its decadal evolution. Over the past decade, global research consortia spanning neuroscience, materials science, artificial intelligence, and clinical medicine have generated a highly compartmentalized knowledge base [10]. This study proposes to employ co‐occurrence network analysis and citation path analysis to objectively trace the evolution from fundamental neural mechanisms to translational applications, elucidating synergistic‐competitive dynamics among technological pathways. Through multi‐dimensional bibliometric indicators—including national/institutional collaboration networks, co‐citation mapping, and keyword evolution—we aim to delineate the global research landscape of BCIs in neuroscience. This approach will reveal endogenous innovation drivers and technology diffusion patterns, provide quantitative evidence for addressing core challenges and optimizing research ecosystems, and ultimately catalyze the transition of BCIs from laboratory prototypes to broad clinical deployment—fulfilling their potential to revolutionize neurorehabilitation.

## 2 Methods

### 2.1 Data Sources and Search Strategy

**FIGURE 1:**
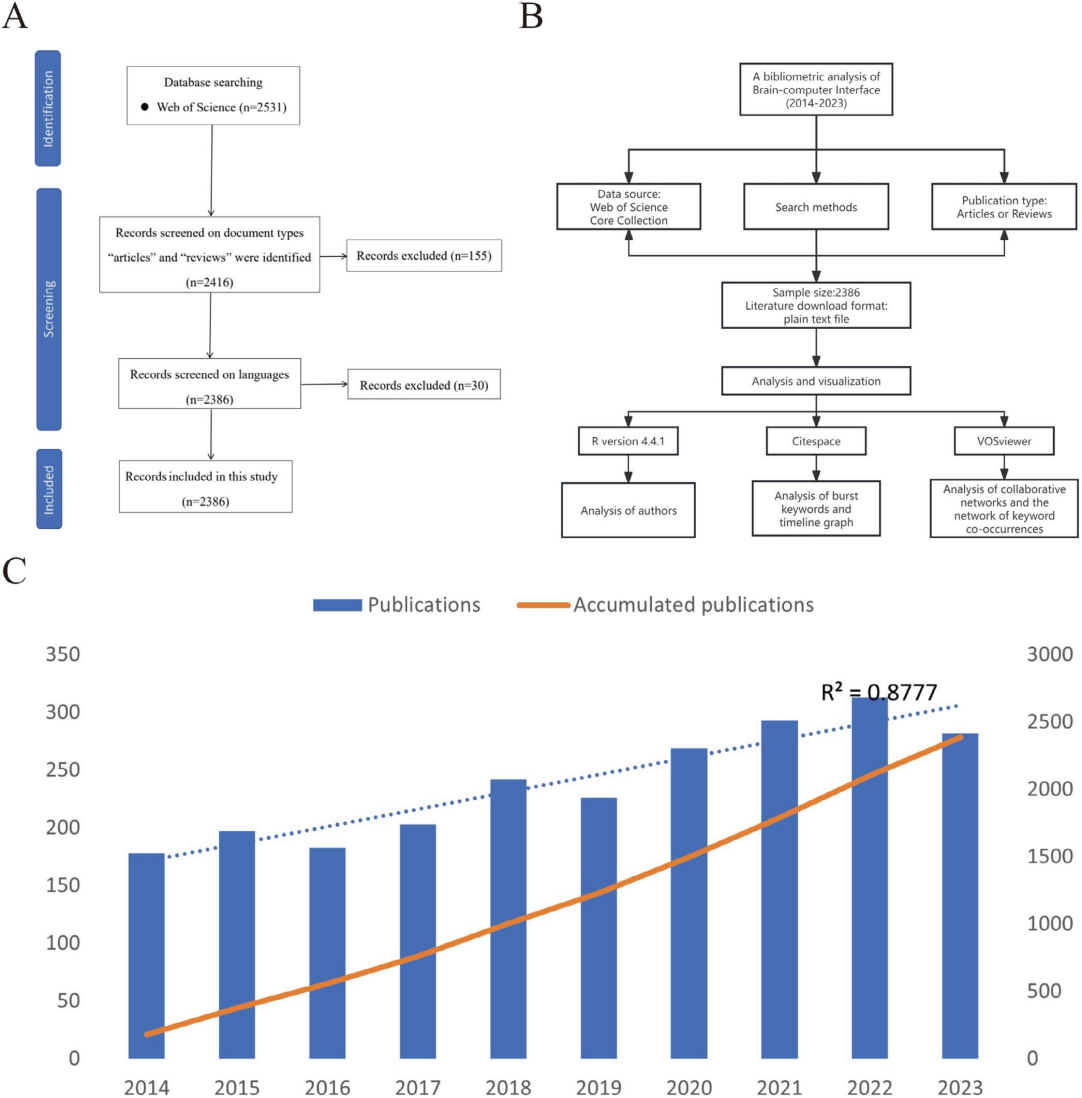
General outline of the study. (A) The process of inclusion and exclusion of documents; (B) Flowchart of the screening process. (C) The number of publications by year over the past 10 years.

Compared to other databases such as Scopus and PubMed, Web of Science offers the most comprehensive and reliable bibliometric analysis [11]. As shown in Table 1, the analysis is based on publications from the Web of Science Core Collection from 2014 to 2023. The relevant publications in the Web of Science Core Collection were searched on 6 August 2024. We developed a search strategy while reviewing previously identified relevant literature. Our search utilized the following keywords: “brain‐computer interface”, “brain‐machine interface”, “brain computer interface”, “brain machine interface” or “direct neural interface”. Inclusion criteria were limited to studies that (1) involved BCI technologies and (2) were related to neuroscience. Exclusion criteria were also defined with source types and the language of the studies. The process of inclusion and exclusion of documents can be seen in Figure 1A. All the retrieved literature was independently reviewed and validated by two researchers to guarantee the pertinence of the papers to the research subject. In instances of disagreement, a third expert was consulted for resolution.

**TABLE 1:** Summary of data source and selection.

Data source	Web of Science core collection
Citation indexes	SCI‐EXPANDED
Searching period	2014 to 2023
Search terms	TS = (“brain‐computer interface” or “brain‐machine interface” or “brain machine interface” or “brain computer interface” or “direct neural interface”)
Subject categories	“Neurosciences”
Document types	“Articles” or “reviews”
Language	“English”
Sample size	2386

Abbreviation: TS, Topic.

### 2.2 Analysis Tools

1. VOSviewer, a cost‐free Java application designed for document mapping, developed by the Centre for Science and Technology Studies at the University of Leiden in the Netherlands, was employed in this research to examine collaborative networks as well as the network of keyword co‐occurrences [12].
2. Citespace, a scientometric tool developed by Chaomei Chen, was used to analyze burst keywords and timeline graph in this study [13].
3. R version 4.4.1, a significant update to the R programming language, is enhancing performance and introducing new features for data analysis and visualization. It was employed to make a bubble chart of authors [14, 15].

### 2.3 Data Analysis

The analytical tools in the Web of Science database were utilized to compile external attributes, such as the count of publications and the average citation count. Bibliographic records along with citation reference information were exported in plain text format.

## 3 Results and Discussion

### 3.1 Overview of Publication Status

As shown in Figure 1B, based on the inclusion and exclusion criteria, a total of 2386 conventional articles on BCIs in the field of neuroscience were included in this study. Figure 1C illustrates the annual publication count related to BCIs. From 178 publications in 2014 to 282 publications in 2023, the cumulative number has steadily increased. In January 2019, Chmielewski, a participant in a BCI study at Johns Hopkins University, underwent a 10‐h surgery to implant six microelectrode arrays (MEAs) bilaterally in the brain. Subsequently, researchers have been working to refine and train him to gain the ability to simultaneously control two prosthetics. This great process may lead to a rapid increase in publications since 2019. An exponential growth function was used to evaluate the relationship between the annual publication volume and publication year, which aligns with the trend in the number of publications per year (*R* ^2^ = 0.8777). This strong correlation indicates that BCIs have experienced significant growth and development in the field of neuroscience in recent years.

### 3.2 Analysis of Countries/Regions and Institutions

**FIGURE 2:**
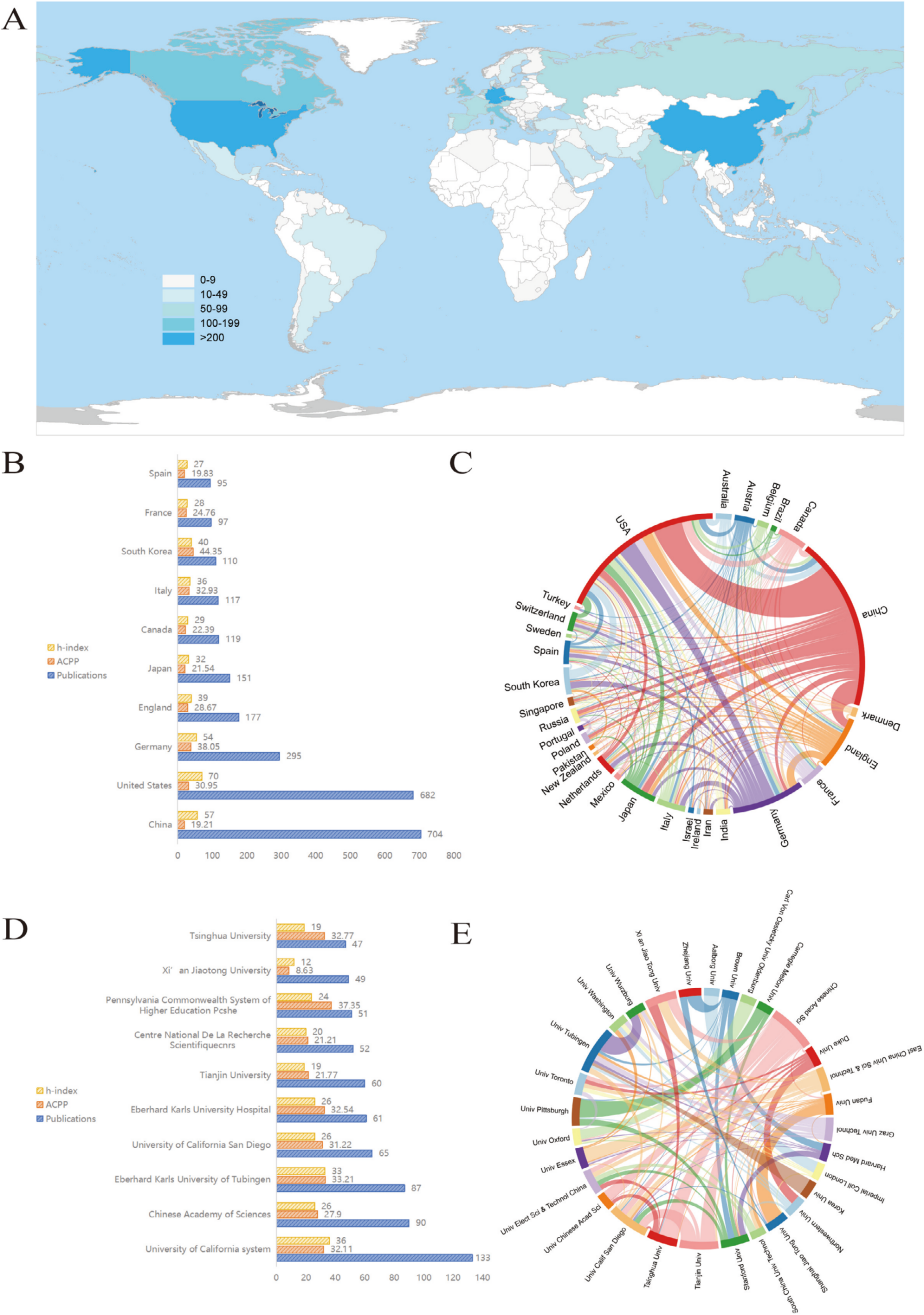
Analysis of related countries/regions and institutions. (A) Each country's contribution to the BCI; (B) Publications in the 10 most productive countries/regions; (C) Collaboration matrix map among the top 30 most productive countries; (D) Publications in the 10 most productive institutions; (E) Collaboration matrix map among the top 30 most productive institutions. TC, total citations; ACPP, average citations per publication.

Nation publication counts were analyzed to investigate the countries/regions contributing the most in this field. A total of 73 countries/regions and 2197 institutions were involved in the publication of 2386 papers. Figure 2A shows each country's contribution to BCIs. The top 10 countries/regions for publications are shown in Figure 2B. ACPP refers to average citations per publication. China had the highest number of publications during the study period (*n* = 704, 29.51%), followed by the United States (*n* = 682,28.58%) and Germany (*n* = 295, 12.36%). China ranked first in terms of article output while its average citation count and h‐index were relatively low. In terms of the number of published papers, the top 10 countries/regions account for more than 90.00% of all papers, indicating an imbalance in the research development among countries/regions.

Figure 2D shows the top 10 institutions in publications within the field. University of California system had the highest number of papers published during the study period (*n* = 133, 5.57%), followed by the Chinese Academy of Sciences (*n* = 90, 3.77%), Eberhard Karls University of Tubingen (*n* = 87, 3.65%), and the University of California San Diego (*n* = 65, 2.72%).

**FIGURE 3:**
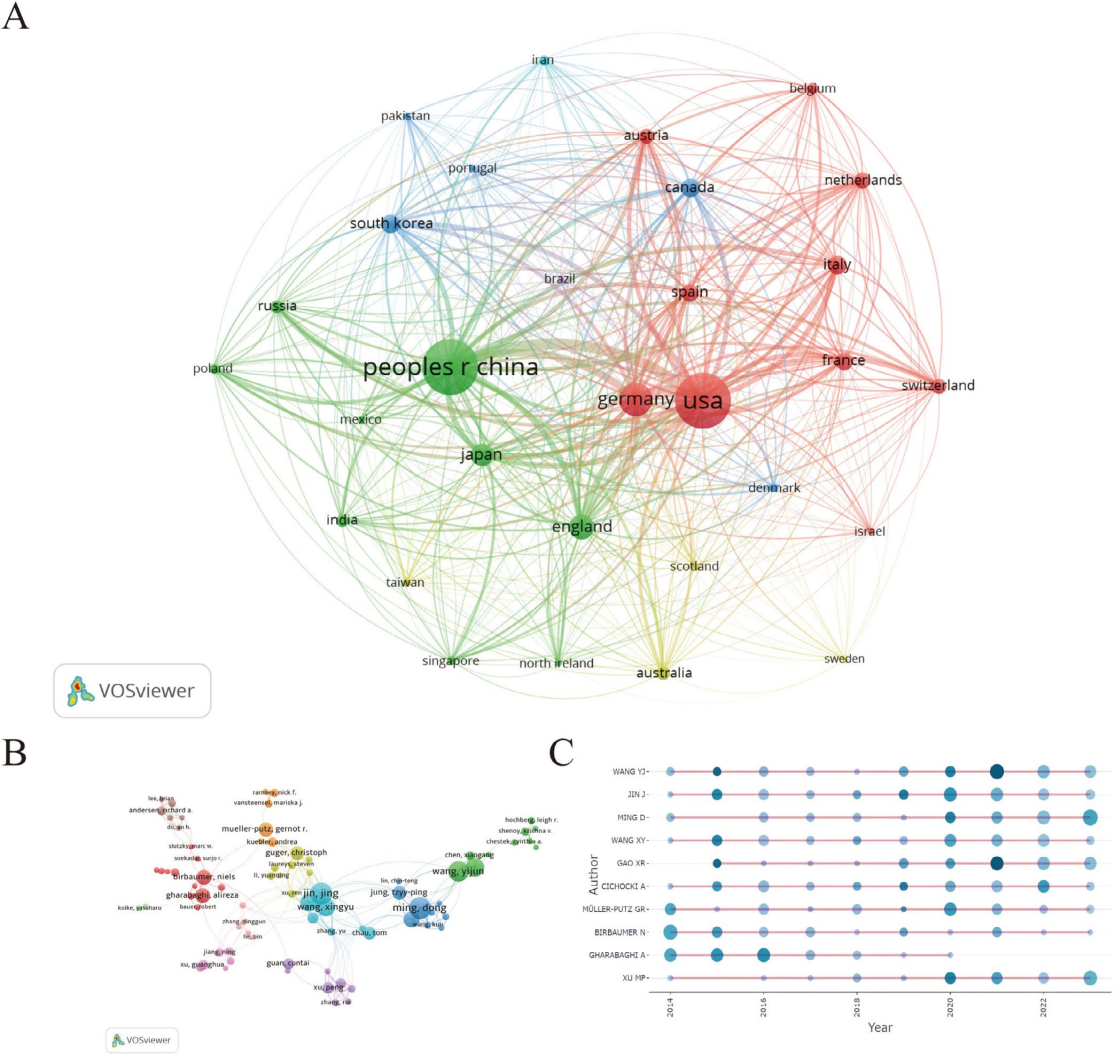
Analysis of related countries/regions and authors. (A) Cooperation intensity among the top 30 countries; (B) The visualization of authors of BCIs in neuroscience; (C) A bubble chart of the top 10 productive authors' production each year.

The study by Wuchty et al. indicates that teams often publish research with greater impact than individuals, and an analysis of the collaborative relationships between different countries/regions, institutions, and authors can also reflect the academic exchange within this field [16]. As part of our investigation, we visualized the collaborations among countries/regions in Figures 2C and 2E. The varying thickness of lines connecting different countries or institutions reflects the level of collaboration between nations, with most countries engaging in cooperation. Notably, the more frequent the exchange between countries or institutions, the higher the output. According to the results, China leads the way in BCI research. The most frequent collaborations are between China and the USA. These two countries play a significant role in the collaborative development within this field. Additionally, Figure 2E illustrates that research institutions from China are collaborating closely with one another, forming a new BCIs research network. Collaboration between nations and institutions is one of the future directions for development. We employed bibliographic coupling analysis in VOSviewer to examine the cooperation intensity among the top 30 countries in terms of publication output and analyzed the substantive impacts arising from such cooperation in Figure 3A. Cooperation between China and the United States in the field of BCIs has promoted technological complementarity. The United States boasts profound accumulations in algorithms and clinical translation, while China has achieved breakthroughs in the research and development of hardware such as flexible neural electrodes. This collaboration has accelerated the maturation of invasive and interventional BCI technologies and facilitated their application and implementation in fields including rehabilitation medicine [17].

### 3.3 Analysis of Journals and Citations

To explore the contributions of various journals to global research on BCIs, we analyzed the number of publications from different institutions. To some extent, journal analysis can help find the core journals in the academic field [18, 19]. The BCI technology has been widely utilized in various domains, encompassing medical, rehabilitation, and entertainment, among others [20, 21, 22]. The analysis reveals that within the duration of our study, 123 journals were involved in the exploration of BCI applications within the field of neuroscience, and the top 10 productive journals are listed in Table 2. The Journal of Neural Engineering published the largest number of publications (*n* = 577, 24.18%) on this topic. The impact factor of this journal is 3.7. It was followed by Frontiers in Neuroscience (*n* = 360, 15.09%), Frontiers in Human Neuroscience (*n* = 269, 11.27%), and the Journal of Neuroscience Methods (*n* = 124, 5.20%). The journal that boasts the highest mean citation frequency is Neuroimage, suggesting that it may be a leading or authoritative journal in the field.

**TABLE 2:** Publications in the 10 most productive Journals.

Rank	Journal	Publications	TC	ACPP	IF	h‐index	Country
1	Journal of Neural Engineering	577	17,765	30.79	3.7	64	UK
2	Frontiers in Neuroscience	360	6567	18.24	3.2	42	Switzerland
3	Frontiers in Human Neuroscience	269	6565	24.41	2.4	43	Switzerland
4	Journal of Neuroscience Methods	124	3135	25.28	2.7	32	Netherlands
5	Brain Sciences	81	1192	14.72	2.7	17	Switzerland
6	Neuroimage	67	2274	33.94	4.7	29	United States
7	Journal of Neuroengineering and Rehabilitation	57	1806	31.68	5.2	26	UK
8	Cognitive Neurodynamics	52	854	16.42	3.1	17	Netherlands
9	IEEE Transactions on Cognitive and Developmental Systems	42	1039	24.74	5.0	17	United States
10	Frontiers in Neurorobotics	41	1197	29.2	2.6	17	Switzerland

Abbreviations: ACPP, average citations per publication; IF, impact factor; TC, total citations.

### 3.4 Analysis of Authors

Analyzing authors can reveal the principal contributors in a field, and to some extent, reflect the research trends within that domain. The h‐index is a blended quantitative measure employed to assess the scholarly accomplishments of researchers. It signifies that an individual has ‘h’ publications, each cited at least ‘h’ times during a specified timeframe. A higher h‐index denotes a more significant influence on academic research [23]. A total of 8043 authors have participated in related studies in the past 10 years. Table 3 lists the top 10 authors by the number of publications. Wang YJ takes the lead in the list with a total of 40 and his h‐index is 19. The following authors are Jin J, Ming D, and Gao, Xiao. Regarding the h‐index, Gharabaghi A ranks first with 22, followed by Wang YJ, Müller‐putz GR, and Birbaumer N, which indicates that they are influential in this field.

**TABLE 3:** Publications in the 10 most productive authors.

Rank	Author	Publications	TC	ACPP	h‐index
1	Wang YJ	40	1509	37.73	19
2	Jin J	39	1304	33.44	16
2	Ming D	39	707	18.13	14
4	Wang XY	34	1016	29.88	16
5	Gao XR	33	1270	38.48	15
6	Cichocki A	32	1181	36.91	16
7	Müller‐putz GR	30	949	31.63	17
8	Birbaumer N	29	903	31.14	17
9	Gharabaghi A	29	1106	38.14	22
10	Xu MP	29	574	19.79	13

Abbreviations: ACPP, average citations per publication; TC, total citations.

We have constructed a collaborative network that includes 79 authors, each with at least 10 relevant publications (Figure 3B). This is a graph obtained using VOSviewer's clustering algorithm. From the graph, we can observe the close collaborations among different authors; for instance, Jin Jing collaborates with Wang Xingyu, Zhang Yu, and Chau Tom, as indicated by the blue lines.

Figure 3C illustrates a bubble chart of the top 10 productive authors' production each year. The size of the circle and the depth of the color represent the authors' publication volume and the total annual citation count, respectively. It illustrates that from 2014 to 2016, BCIs were widely investigated within the neuroscience domain. Between 2016 and 2019, there was a noticeable decline in the number of publications by authors researching BCIs. Following 2019, these authors have rekindled their research efforts, leading to a resurgence in publications on this topic.

### 3.5 Analysis of Keywords

**FIGURE 4:**
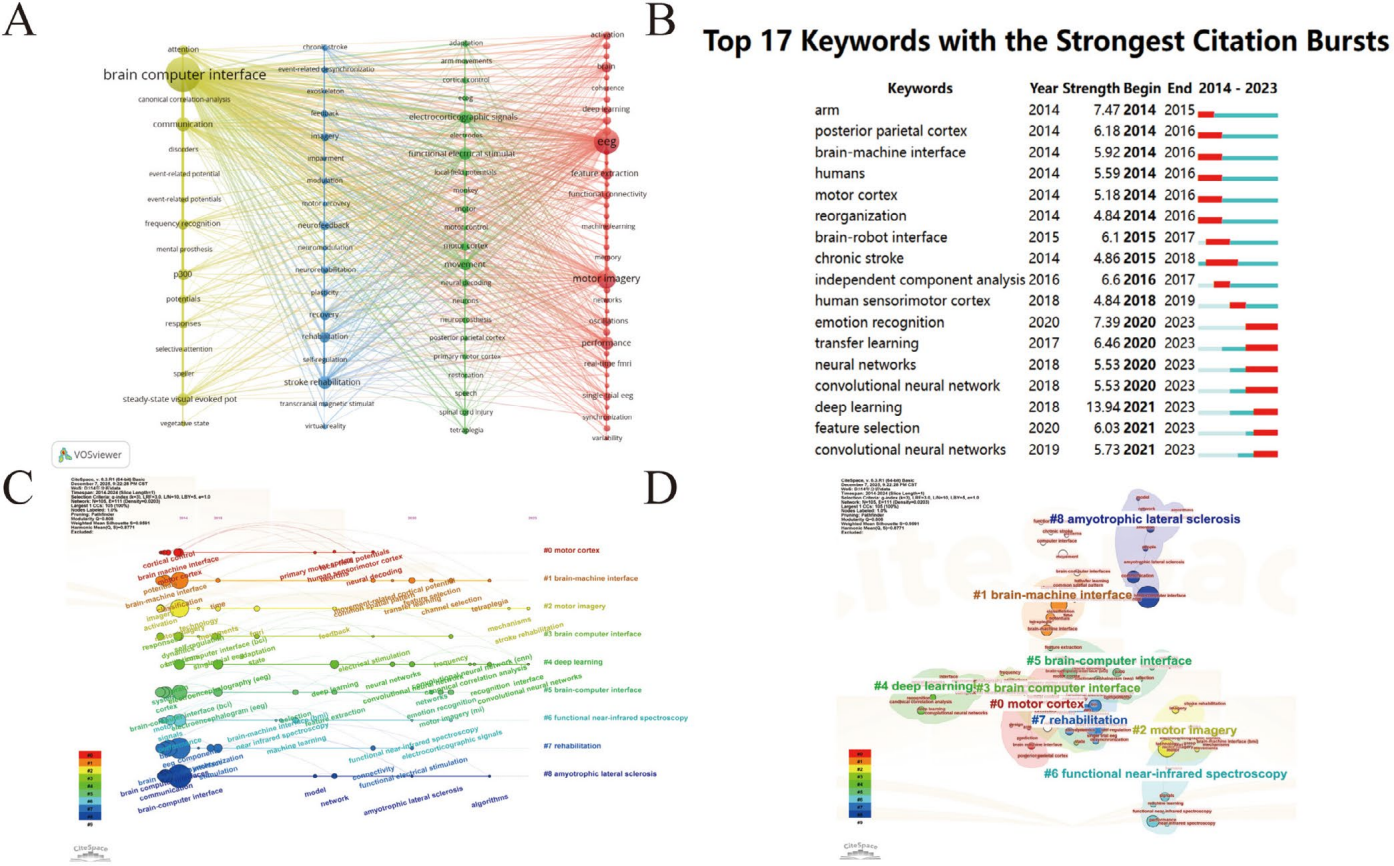
Analysis of keywords. (A) Keyword co‐occurrence network; (B) Top 17 keywords with strong citation bursts between 2014 and 2023; (C) The timeline graph of research hotspots; (D) The keyword cluster map.

From a bibliometric perspective, keyword analysis is beneficial for deepening the knowledge structure within academic fields, which aids in uncovering potential research hotspots [24]. We analyzed keywords that appeared more than 19 times, which was computed based on the clustering algorithm of VOSviewer. The analysis included the titles and abstracts of 2386 papers, from which we extracted the 95 most frequently occurring keywords and vividly represented them. As shown in Figure 4A, the color of the elements indicates the cluster to which they belong. The size of the nodes reflects the frequency of keyword occurrence, while the thickness of the links represents the strength of co‐occurrence. Thicker links between nodes indicate a stronger co‐occurrence between keywords. We used four colors (red, green, blue, and yellow) to divide the keywords into four groups, highlighting mainstream research hotspots and emerging frontiers in the field.

Cluster 1 is focused on Neuroscience. The main keywords were “brain‐computer interface”, “canonical correlation‐analysis”, “event‐related potential”, “frequency recognition”, “p300” and “selective attention”. Cluster 2 is primarily associated with Neurorehabilitation. The primary keywords were “chronic stroke”, “event‐related desynchronization”, “exoskeleton”, “motor recovery”, “neuromodulation”, “neurorehabilitation” and “stroke rehabilitation”. Cluster 3 is related to Motor Control. The keywords were “functional electrical stimulation”, “electrocorticographic signals”, and “motor cortex”. Cluster 4 is focused on Cognitive Neuroscience. The essential keywords were “EEG”, “therapy”, “single‐trial EEG”, “deep learning” and “functional connectivity”. These 4 themes constitute the mainstream academic literature on BCIs in Neuroscience.

By using CiteSpace's keyword burst detection algorithm, we can accurately identify high‐frequency keywords over specific periods, offering insights into research trends in the field. Our analysis highlights the 17 keywords with the most notable burst activity. The result is shown in Figure 4B. “Deep learning” shows the highest burst intensity (13.94), followed by “emotion recognition” (7.39) and “arm” (7.47). The earliest bursts were noted for keywords like “arm”, “posterior parietal cortex”, “motor cortex”, “humans” and “reorganization” signifying early research hotspots. Later, “independent component analysis” and “human sensorimotor cortex” emerged as key research topics. Recent research directions include “deep learning”, “feature selection” and “convolutional neural network” reflecting the latest areas of focus.

The timeline graph shows how research hotspots, represented by keywords, have evolved over time and explores the relationships between clusters. The number of documents in each cluster indicates the depth and importance of the research. A network was created using CiteSpace parameters, consisting of 101 nodes, 111 connections, and a density of 0.0203. This visualization emphasizes the main areas and emerging trends in brain‐computer interfaces within neuroscience. Cluster IDs are numbered sequentially (e.g., #0, #1, #2). Figure 4C shows nine distinct clusters, including motor cortex, brain‐machine interfaces, motor imagery, brain computer interface, deep learning, brain‐computer interface, functional near‐infrared spectroscopy, rehabilitation, and amyotrophic lateral sclerosis. Then, we generated a keyword clustering map, and eight clusters were generated. Modularity (Q) and silhouette (S) are crucial indicators for evaluating the clustering level. Generally, the Q greater than 0.3 and S greater than 0.5 indicate a reasonable cluster structure and convincing results. After the clustering, the following parameters were obtained: Q = 0.8054 > 0.30 and S = 0.8766 > 0.5, indicating reasonable clustering and high confidence.

In the keyword cluster map, we display 9 large‐scale clusters. The clustering labels start from 0; the smaller the value, the larger its size. The three most significant clusters are #0 brain‐machine interface, #1 deep learning, and #2 brain‐computer interface. We further combined the cluster names and browsed the keywords contained in the eight clusters to further summarize the eight clusters into three main clusters, namely cluster 1 (#0 brain‐machine interface, #2 brain‐computer interface): the research into the core elements of the entire brain‐computer interface chain. Cluster 2 (#4 motor imagery and #5 stroke): the application and mechanism research of brain‐computer interface technology based on motor imagery in stroke rehabilitation, and cluster 3 (#1 deep learning, #3 motor cortex, #6 electroencephalogram, #affective brain‐computer interface and #8 machine learning): the research into the technology and applications of motor cortex and emotional brain‐computer interfaces (Figure 4D).

## 4 Discussion

### 4.1 General Finding

Bibliometric methods were used to analyze the growth pattern of BCI research from 2014 to 2023. In general, the research on BCIs has been increasing in the past 10 years. By 2023, the annual number of relevant papers has exceeded 250, indicating that the research on BCIs has entered a rapid development mode.

China and the United States dominate the number of publications in 10 countries/regions. In addition, the cooperation between China and the United States is dominant among the 30 countries. The above findings confirm the key contribution and leading position of China and the United States in BCI research, which may be due to the national economic conditions and high level of medical investment in China and the United States. This field will benefit from extensive international cooperation, which will improve the overall level of research.

### 4.2 Hotspots and Frontiers

Analysis of high‐frequency keywords reflects the hotspots in a particular research field. We used key co‐occurrence analysis to determine the main directions and hotspots, as well as to uncover the development and changes of its theme structure [25]. Cluster analysis based on keywords finally formed a cluster of four colors. Then, according to the analysis of the top 17 keywords with the strongest citation bursts, the research hotspots and frontiers in BCIs in Neuroscience are determined, and its main contents are as follows:

### 4.3 EEG and BCIs

EEG‐BCI detect weak electrical signals produced by synchronized neuronal firing in the cerebral cortex via scalp electrodes. Its methodology centers on two neural markers: event‐related potentials (ERPs) and sensorimotor rhythms (SMR) [26]. Through a pipeline of signal acquisition, preprocessing, feature extraction, and classification, specific control commands are generated. As a representative non‐invasive BCI, EEG‐BCI is predominant in neurofeedback, fundamental brain research, assistive communication, and game control applications, owing to its high temporal resolution, low cost, and portability—a distribution of application scenarios that is also reflected in literature trends, which show a sustained upward trajectory of publications focusing on non‐invasive BCI usability in clinical and daily‐life settings over the past decadey [27].

Over the past decade, EEG‐BCI research has primarily focused on two key areas. First is innovations in signal acquisition technology. Traditional wet electrodes necessitate complex skin preparation prior to use, requiring significant time and inducing participant discomfort [28]. Excessive conductive gel also risks inter‐electrode bridging [29, 30]. This limitation has been a recurrent topic in high‐citation EEG‐BCI studies, as reflected in literature trends that highlight a surge in publications addressing electrode‐skin interface optimization—a critical bottleneck distinguishing non‐invasive EEG‐BCI from high‐spatial‐resolution invasive BCIs such as intracortical microelectrode arrays.

To address these limitations, multiple dry electrode systems have been developed in recent years. Haueisen et al. [31, 32] describe the design of a novel 24‐pin dry electrode made from polyurethane and coated with Ag/AgCl. This system facilitates reliable EEG recording without skin preparation or conductive gel. Lacking conductive gel, microneedle arrays must traverse the hair barrier for direct scalp contact, compromising user comfort [33]. In practical applications, stiffer electrode materials can more readily penetrate the hair layer to maintain stable scalp contact while reducing motion artifacts; conversely, more flexible materials enhance comfort [34].

To resolve this trade‐off, Jin et al. [35] synthesized an adhesive polydimethylsiloxane (aPDMS) self‐adhesive conductive composite by uniformly blending carbon nanotubes (CNTs) with end‐methylated polysiloxane, combining advantages of dry and wet electrodes. This material yielded high‐quality EEG signals in human fibroblast and skin tests. Given direct skin interfacing of microneedle electrodes, appropriate materials are essential to minimize interfacial resistance [36]. Li et al. [37] developed a semi‐flexible PDA/Pt‐TiO_2_ dry electrode for SSVEP‐BCI systems, where synergistic effects of PDA biofilm adsorption, Pt NP electron trapping, and TiO_2_ NT electron transfer lowered electrode‐scalp contact resistance. This increased recorded EEG signal amplitude, demonstrating significant application potential. However, widespread practical implementation of dry electrodes remains challenged by factors including poor contact noise, difficulties in maintaining stable electrode connections, high costs, and other limitations [38].

The second focal area identified by literature analysis, which directly links algorithmic advances to the resolution of non‐invasive BCI's inherent signal limitations, is progress in signal decoding algorithms; advances in signal decoding algorithms have broadened EEG‐BCI applications. Characterized by physiological signal attenuation and noise contamination, EEG signals inherently possess a low signal‐to‐noise ratio and limited spatial resolution [39]. The algorithmic revolution driven by machine learning—particularly deep learning—has significantly improved decoding accuracy across spatial–temporal dimensions [40].

As the fundamental unit of deep learning, artificial neurons apply nonlinear transformation to the linear combination of its inputs, obtaining the high‐level features. Stacking these neurons in different ways, a variety of deep learning frameworks are built to implement effective feature extraction. Convolutional Neural Networks (CNNs) represent the predominant framework for EEG processing [41]. In 2015, Schirrmeister et al. [42] pioneered the use of a 13‐layer CNN for raw EEG analysis, achieving significantly higher accuracy in decoding pathological EEG outcomes. For epilepsy detection, this approach demonstrated a 6.9% accuracy improvement over SYM and CSP methods. CNNs are also typically integrated with other architectures for emotion recognition tasks, with applications spanning scenarios such as driver fatigue monitoring. However, high inter‐subject variability in EEG signals necessitates critical consideration of generalization capabilities in deep neural network design. CNNs' dependence on extensive subject‐specific parameters limits their generalizability across paradigms. In contrast, the specialized EEGNet architecture substantially reduces parameter counts while increasing cross‐subject accuracy from 51% to 68%.

Long Short‐Term Memory (LSTM) networks, widely employed in modeling natural language [43], image [44], video [45], and speech data [46], have been initially explored for EEG sequence processing. A hierarchical LSTM (H‐LSTM) model proposed by Hasib et al. [43] encodes local‐temporal correlations among EEG samples within segments at its first layer and temporal dependencies between segments at the second layer. This approach effectively predicts human decisions in relevant applications. While such networks are commonly deployed on desktop or cloud platforms, persistent latency and data privacy concerns associated with remote computing remain. Processing data on low‐power Microcontroller Units (MCUs) mitigates these issues to some degree. However, computationally intensive models like TPCT—with 7.78 million trainable parameters and 1.73 billion Multiply‐Accumulate (MAC) operations per inference—exceed typical MCU constraints [47]. To concurrently achieve high accuracy and model compactness, EEG‐TCNet, introduced by Thorir et al. [48], enables lightweight deployment via parameter quantization and knowledge distillation while preserving computational efficacy.

In summary, literature trends in EEG‐BCI research not only mirror the field's technical evolution but also highlight the key neuroscientific and clinical challenges that distinguish non‐invasive from invasive BCI approaches. EEG‐BCI research has now entered a new phase. While numerous technologies have proliferated, several critical challenges remain unresolved. For instance, EEG signals exhibit low signal‐to‐noise ratios and inferior spatial resolution compared to fMRI and fNIRS. This necessitates the development of enhanced multimodal integration paradigms to reduce motor decoding error rates. Future research should prioritize developing deep learning approaches capable of handling the high interdisciplinary variability inherent in EEG signals, thereby improving the clinical translatability of neural network models.

### 4.4 Neural Rehabilitation and BCIs

Literature metrology trends over the past two decades consistently highlight neural rehabilitation as the highest‐citation subfield of BCI research, a trend that directly reflects the translational urgency of addressing motor impairment—a core clinical challenge where both invasive and non‐invasive BCI approaches compete and complement each other [49]. BCI technology is often used to induce neuroplasticity in the brain and help patients regain motor function. In particular, great progress has been made in helping disabled people control prosthetics [50], propel wheelchairs [51], drive vehicles [52], and even spell, type, and play online games [53]. BCI systems have demonstrated significant translational potential in neural rehabilitation, particularly for motor function recovery in stroke survivors and symptom management in multiple sclerosis (MS) patients [54, 55].

Stroke is a sudden and localized cerebral dysfunction. It imposes a significant societal burden and severely impacts quality of life [56, 57]. Keywords analysis identifies stroke as the most studied clinical indication for BCI rehabilitation, with a growing number of publications focusing on closed‐loop BCI‐neurostimulation paradigms—a shift that responds to the key challenge of enhancing neuroplasticity in damaged motor cortices, a limitation that early open‐loop BCI systems failed to address. Several studies have explored the safety, feasibility, and clinical efficacy of BCIs for rehabilitating limb motor function after a stroke. Buch was the first to apply the BCI system to stroke rehabilitation, involving eight chronic stroke patients who completed over 20 rehabilitation sessions using the EMG‐BCI system [58]. Although this study confirmed the feasibility of BCI control in stroke patients, it did not show significant clinical improvements.

The first successful clinical improvement was reported by Ang et al. In their study, eight chronic stroke patients underwent BCI‐mediated upper extremity rehabilitation [59]. After 12 sessions over 4 weeks, the patients exhibited significant improvements in motor function. Conversely, Ramos‐Murguialday validated the effectiveness of BCI intervention through a randomized controlled trial, highlighting the crucial role of systematic and accurate feedback [60]. The study also found that various combinations of rehabilitation techniques had a cumulative effect, proving particularly beneficial for upper limb rehabilitation.

MS is a debilitating chronic illness in which the immune system erroneously attacks the myelin sheath that protects nerve fibers in the central nervous system. Recent studies indicate that BCIs hold promise as an alternative treatment for MS, helping patients regain independence and control. Previous research has shown that BCIs can enhance autonomy for MS patients, particularly during periods of fatigue, and function as a hybrid control system for neural activity [61]. For instance, a BCI equipped with a visual guidance system, as studied by Downey et al., allows quadriplegics to use prosthetic limbs for grasping tasks effectively [62]. Moreover, other research has found that BCIs can be integrated into rehabilitation programs that utilize neuromuscular electrical stimulation [63]. These rehabilitative BCIs can promote neuroplasticity by connecting neural prostheses to damaged areas or by reactivating impaired synaptic networks [64]. An example is the modular assistive motor framework designed by Alessandra et al., which leverages a patient's remaining motor abilities to facilitate interaction with their environment [65].

The development and application of BCIs for MS are still in the early stages. However, a marked gap persists between the expanding body of foundational BCI research in MS and the scarcity of long‐term clinical trials, a divergence that underscores the translational challenges of assessing BCI efficacy in heterogeneous, progressive patient populations with variability far exceeding that of stroke cohorts, highlighting the need for further investigation into this population [66, 67].

### 4.5 Limitation

We must acknowledge the limitations of this study. First, our analysis focused exclusively on the sci‐expanded 1999‐present database within the Web of Science Core Collection. This database includes scientific literature from 1999 to the present and offers comprehensive indexing, which aids users in understanding research developments and citations across various fields. However, limiting our search to just one database may have led to missing some significant findings. Second, while CiteSpace, R, and VOSviewer assist in visually analyzing citation and keyword networks, they may not provide a complete overview of the publications. Third, limitations in our expertise may mean that our self‐designed keyword‐counting process is insufficient. In the future, we plan to expand our data sources, refine our analysis methods, and standardize the keyword screening process to enhance the accuracy of our findings.

## 5 Conclusion

In this study, we meticulously investigate and present the research process and development achievements of BCIs from 2014 to 2023. The study analyzes influential researchers, institutions, and countries involved in BCI research. The findings indicate that China and the United States are highly influential in this field, while there is a research imbalance among other countries and regions. Therefore, strengthening cooperation and communication between different countries/regions and institutions will facilitate further development in this domain. In recent years, the research focus in this field has primarily been on neuroscience, neurorehabilitation, motor control, and cognitive neuroscience. This study provides researchers with detailed information needed to understand the current state, collaboration networks, and major research hotspots in the field while elaborating on the evolution and frontiers of this domain.

## Author Contributions

Yangfan Yu and Wentong Liu designed the study and wrote the manuscript. Sirui Ju, Lishan He, Nanting Chen, and Alexandr N. Chernov analyzed the data. Jianfeng Zhang, Jinning Mao, and Guodong Liu revised the manuscript.

## Funding

This study was not supported by any funding programs.

## Conflicts of Interest

The authors declare no conflicts of interest.

## Supporting information


Data S1.

## Data Availability

The data that supports the findings of this study are available in the Supporting Information of this article.
